# Efficacy of Anthelmintics Against Canine Hookworm Infections in the Bono East Region of Ghana

**DOI:** 10.1155/japr/4079763

**Published:** 2025-02-19

**Authors:** Samuel Ayetibo Ofori, Papa Kofi Amissah-Reynolds, Opoku Gyamfi, Kofi Agyapong Addo, Simon Nyarko, Victor Agyei, Joshua Dwomoh, Esther Ayemugah

**Affiliations:** ^1^Department of Biological Sciences Education, Akenten Appiah-Menka University of Skills Training and Entrepreneurial Development, Ashanti Mampong, Ghana; ^2^Department of Chemistry Education, Akenten Appiah-Menka University of Skills Training and Entrepreneurial Development, Ashanti Mampong, Ghana; ^3^Department of Pharmaceutics, Kwame Nkrumah University of Science and Technology, Kumasi, Ghana; ^4^Department of Public Health Education, Akenten Appiah-Menka University of Skills Training and Entrepreneurial Development, Ashanti Mampong, Ghana

**Keywords:** albendazole, biochemical and hematological parameters, drug resistance, niclosamide, pyrantel

## Abstract

Hookworm infections present a major health risk to dogs, especially in areas characterized by warmer climates and poor sanitation. This cross-sectional study was undertaken to determine the prevalence of hookworm infections and the efficacy of anthelmintic treatments in dogs from the Bono East Region of Ghana. Four hundred and ninety-one (491) canine stool samples were examined using the McMaster technique to ascertain the prevalence of hookworms. Using in vivo and in vitro techniques, the efficacy of three anthelmintics (albendazole, pyrantel, and niclosamide) was assessed in an experimental control trial involving dogs naturally infected with hookworms. The effects of the drugs on hematological and biochemical parameters were measured within a 14-day period to assess changes over time. The study found a total prevalence of 54.2% (266), with significantly higher infection rates in puppies (69.8%, 97), hunting dogs (64.1%, 91), and rural dogs (84.2%, 160). Logistic regression identified age, purpose, and settlement type as risk factors for infection. Of the three treatments, niclosamide was the most efficacious, reducing egg counts by 95%, while albendazole was the least efficacious (−69%). In vitro tests confirmed the superior performance of niclosamide, with the lowest IC_50_ value of 29.19 *μ*g/mL. Hookworm-infected dogs exhibited anemia, eosinophilia, hypoalbuminemia, and hypoproteinemia. There was significant improvement in the hematobiochemical parameters after treatment, particularly in niclosamide-treated dogs. Veterinarians can consider niclosamide, especially in resource-limited settings, due to its affordability. The findings emphasize the importance of regular monitoring and treatment of hookworm infections to improve the overall health and well-being of dogs in the region. Herein, we report for the first time on reduced efficacy of albendazole and pyrantel against dog-related hookworms in Ghana.

## 1. Introduction

Hookworm infections are major health issues for companion animals, particularly dogs and cats, across the globe [1], and Ghana is no exception. These infections caused by parasitic nematodes can lead to various pathological consequences and pose a threat to the overall well-being of dogs. Several studies have documented hookworm infections in dogs from various regions, including Bangladesh [2], Australia [3], India [1, 4], Nigeria [5, 6], and Ghana [7–9], highlighting the widespread nature of hookworms and the potential zoonotic risk to humans. The presence of hookworm species such as *Ancylostoma caninum*, which is also found in humans, suggests a possible role of dogs as reservoirs for zoonotic hookworm infections [10, 11]. The high prevalence of hookworms in dogs and the associated public health risks emphasize the importance of effective anthelmintic treatment strategies [12, 13].

Anthelmintics are commonly used for the treatment and control of hookworm infections. These include pyrantel (PP) pamoate, ivermectin, fenbendazole, milbemycin oxime, and benzimidazole (BZ) drugs such as albendazole (ABZ), levamisole, and mebendazole [14]. These drugs have largely been efficacious in combating hookworm infections, helping to reduce parasite burden and improving the overall health of infected dogs. However, the emergence of anthelmintic resistance in hookworms is a growing concern globally [12, 15, 16]. Anthelmintic resistance occurs when hookworm populations become less susceptible or entirely unresponsive to the activity of anthelmintic drugs. This resistance can be attributed to several factors, including genetic characteristics of the parasites, selection pressure from repeated anthelmintic treatments, and environmental factors [17]. The consequences are dire, leading to treatment failures, prolonged infections, and potential negative impact on animal health.

In the United States, there have been persistent reports of hookworm infections in dogs, despite treatment with anthelmintics. Resistance has been observed across multiple breeds, with greyhounds being significantly overrepresented [10]. The development of real-time PCR assays has facilitated the detection of the F167Y polymorphism, a genetic marker associated with BZ resistance, in *A. caninum* [17]. In Canada, the presence of genetic BZ resistance markers in *A. caninum* has also been reported, indicating the emergence and potential spread of resistant hookworms within the country [18]. The issue of anthelmintic resistance is not restricted to North America. In the Pacific region, a study reported significant reduction in the efficacy of PP embonate against *A. caninum*, suggesting high-level anthelmintic resistance [19]. Also, the efficacy of levamisole has been found to vary with age, thus necessitating strict adherence to recommended treatment regimens [20].

Apart from anthelmintic resistance, studies have also assessed the effect of hookworm infections on hematobiochemical parameters in dogs [21–23]. In particular, *A. caninum* infections have been linked with hematological changes including red blood cell (RBC) count, hemoglobin (Hb), packed cell volume (PCV), and eosinophil counts [21–24]. Likewise, hookworm-related changes in serum biochemistry, including total protein levels, aspartate transaminase (AST), alanine transaminase (ALT), and alkaline phosphatase (ALP), have also been observed [24, 25].

Recently, there has been a significant increase in the number of household dogs in Ghana [9]. This rise has been accompanied by high prevalence of hookworm infections among dogs [26]. With the global surge in anthelmintic resistance, it has become imperative to assess the situation in Ghana. It is plausible that these dogs are highly exposed to hookworm infections and could be developing resistance to current treatments. Addressing these issues is important for achieving Sustainable Development Goal (SDG) 3, which focuses on healthy lifestyles and the promotion of well-being for all. Therefore, this study sought to assess the efficacy of anthelmintics against canine hookworm infections in the Bono East Region of Ghana.

## 2. Materials and Methods

### 2.1. Study Area

**Figure 1:**
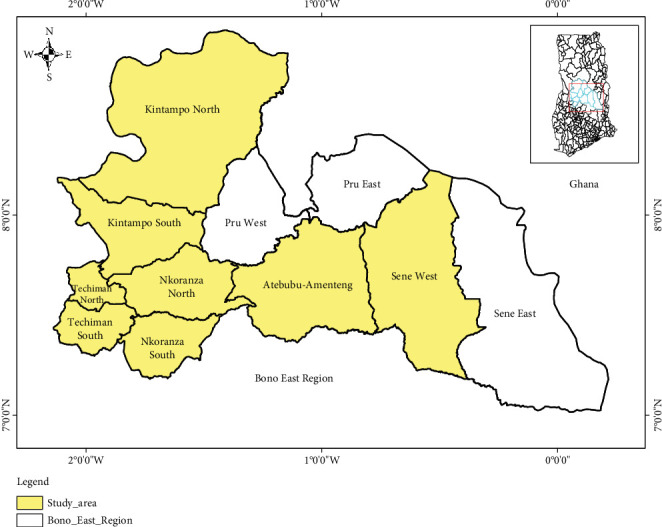
Map of Bono East indicating the various study districts (source: designed using ArcGIS 10.7).

The study was conducted in Bono East Region of central Ghana. The region was created in 2019 following the division of the Brong-Ahafo Region. It is bordered by the Savannah Region to the north, the Bono Region to the west, the Ashanti Region to the south, and the Volta Lake to the east. The region lies within a vegetative zone characterized by consistently favorable climatic conditions. The predominant vegetation in this area consists of forests and fertile soils. The dry season typically occurs from December to April. The wet season generally spans from July to November, with an average annual rainfall ranging from 750 to 1050 mm. The highest temperatures are experienced toward the end of the dry season, while the lowest temperatures occur in December and January. However, the dry and hot periods which fall between December and early February are often accompanied by the hot Harmattan wind originating from the Sahara. During this time, temperatures can fluctuate between 14°C (59°F) at night and 40°C (104°F) during the day. The region is characterized by a mix of agricultural activities, including livestock farming, and is home to several urban centers such as Techiman, Nkoranza, Kintampo, and Atebubu. The study was conducted in eight (8) out of the eleven (11) districts in the Bono East Region of Ghana, excluding Pru West, Pru East, and Sene West (Figure 1) [27].

### 2.2. Population and Sample Size

This study focused on domestic dogs in the Bono East Region of Ghana. A previous study on hookworms in the area indicated a prevalence rate of 47%, which was used to calculate the sample size [26]. Using this prevalence, the formula *n* = (*z*^2^ × *P* × (1 − *P*))/*d*^2^, was used to determine the sample size, where *n* is the required sample size, *Z* is the *Z* value (1.96 for a 95% confidence level), *P* is the estimated prevalence (0.47), and *d* is the desired precision (0.05). Based on this calculation, the final sample size was set to include a minimum of 301 dogs.

### 2.3. Experimental Design and Animal Selection

The study employed an experimental control trial between January and July 2024. Sampling was done in veterinary facilities, farms, and households in both urban and rural areas to capture the diversity of the districts' socioeconomic and environmental factors. Dogs from various districts in the region with or without a history of anthelmintic usage and known hookworm infections were selected for the study. Hookworm-infected dogs were recruited from each district and grouped into cohorts. Each cohort consisted of three treatment groups (ABZ, PP, and niclosamide (NIC)) and placebo (Table 1). The dogs were recruited based on the presence of hookworm infections detected through initial fecal examinations. All recruitments were done in collaboration with local veterinary authorities across the region.

### 2.4. Anthelmintic Treatment

Dogs were divided into four groups and treated orally with suspensions of ABZ, a BZ derivative (Batch No. F230320, Hebei New Century Pharmaceutical Industry, Shijiazhuang, China), Prazivet Plus (praziquantel—50 mg/ PP pamoate—144 mg/febantel—150 mg) (Batch No. P2-230704, Smith & Kenner Pharmaceuticals, India), NIC (Batch No. NSC-178296, Smith & Kenner Pharmaceuticals, India), or a placebo (control group). Both ABZ and NIC were administered at a single dose of 50 mg/kg body weight of dog, while PP was administered at a dose of 34.4 mg/kg of body weight (i.e., manufacturer's recommended doses for dogs). The efficacy of the drugs was evaluated using both in vivo (the fecal egg count reduction test (FECRT)) and in vitro methods. Egg counts were recorded in each treatment unit before drug administration (Day 0) and on Days 4, 8, and 14 after treatment. The posttreatment egg screening followed the same procedure as the pretreatment. All dogs in the control group received a placebo on Day 0. In adherence to ethical considerations, they were treated at the end of the study. At the beginning of the study, the weight of the dogs was recorded, and the dosage of the anthelmintic drug was adjusted based on the instructions provided by the manufacturers.

### 2.5. In Vivo—Fecal Egg Count (FEC) Using McMaster

FECs were done in triplicate using the McMaster technique to process the fecal samples and quantify the eggs (28) as previously described by Paras et al. [28]. Briefly, a 3g fecal sample was measured and placed in a container. Then, 28 mL of NaCl flotation solution was added to the container, mixed, and allowed to soak for 5 min, following the procedure outlined by Paras et al. [28]. The mixture of feces and flotation medium was stirred and strained into another container. Both chambers of the McMaster slide were filled with the mixture of feces and flotation solution using a pipette. The slide was then observed under the microscope using 40 × and 10 × objective lenses. All eggs inside the grid of the slide were counted using the 10 × objective lens, including those on the grid lines if they exceeded half the number of eggs inside the grid in both chambers. The number of eggs in both chambers was multiplied by 50 to obtain the number of eggs per gram (epg) [29]. The efficacy of various postfecal treatments was evaluated using the percentage reduction in FEC. The reduction was calculated using the formula:

 $$\begin{array}{c} \text{FECR}\left(\%\right)=\frac{\left(\text{average egg count pretreatment}\right)-\left(\text{average egg count posttreatment}\right)}{\left(\text{average egg count pretreatment}\right)}\times 100, \end{array}$$

where pretreatment is the arithmetic mean of the epg before the treatment and posttreatment is the arithmetic mean of the epg after the treatment [29]. According to Coles et al. [30], resistance is present if either the percentage reduction in egg counts is less than 95% or the lower 95% confidence limit is less than 90% [30].

### 2.6. In Vitro—Egg Hatch Assay

Fecal samples were collected from the rectum of dogs and processed using a 1.2 g/mL sodium chloride flotation medium. Approximately 100–200 eggs were added to each well of a 24-well plate. For the stock solutions of PP and NIC, 50 mg of each was dissolved in 100 mL of a 50% dimethyl sulfoxide (DMSO) solution. For the ABZ suspension, a mixture of 10% DMSO and distilled water in a 40:60 ratio was used. Serial dilution was performed with distilled water to produce five final concentrations of 0.025, 0.05, 0.065, 0.08, and 1.0 *μ*g/mL in 1% DMSO for each drug. The liquid-based method was used for these assays, and 24-well plates were utilized with minor modifications from a previously described protocol [31]. The final DMSO concentrations were 1% per well for both treatment and control wells, which has previously been shown to be well tolerated by parasites [31]. The experiment was set up to repeat each treatment concentration three times. The last two wells of each row contained control eggs undergoing incubation without drug treatment, while other wells contained increasing concentrations of ABZ, PP, and NIC. The wells were incubated at 42°C–48°C for 48 h, and the number of larvae present in each plate was counted using a microscope. The egg hatch inhibition (EHI) and half-inhibitory concentration (IC_50_) were determined for each concentration of ABZ, PP, and NIC. The EHI was calculated using the formula described by Babják et al. [32]:

 $$\begin{array}{c} \text{EHI }\left(\%\right)=\frac{\left(\text{eggs hatched in the control group}\right)-\left(\text{eggs hatched after drug incubation}\right)}{\left(\text{eggs hatched in the control group}\right)}\times 100. \end{array}$$

### 2.7. Hematological and Biochemical Test

Hematological analysis was performed using blood samples collected with an anticoagulant (EDTA). These samples were then analyzed using Hematology Analyzers (Sysmex XN-1000, Model Number XN-1000, manufacturing in Japan). Hematological parameters include Hb, PCV, total leukocyte count (TLC), differential leukocyte count (DLC), total erythrocyte count (TEC), mean corpuscular volume (MCV), mean corpuscular hemoglobin (MCH), and mean corpuscular hemoglobin concentration (MCHC). Biochemical analysis focused on specific serum parameters, including total protein, albumin, globulin, creatinine, ALT, and AST. For the purpose of this study, the Beckman Coulter AU480 Clinical Chemistry Analyzer (United States) was employed. This model is known for its high throughput, reliability, and precision in biochemical analysis. The blood samples without anticoagulants were allowed to clot, and the serum was separated by centrifugation. The obtained serum samples were stored in appropriately labeled cryovials and maintained at a temperature of −20°C to preserve their integrity prior to biochemical analysis. The serum samples were analyzed using Clinical Chemistry Analyzers, employing specific assays to measure the biochemical parameters accurately.

### 2.8. Data Analysis

Descriptive statistics was used to determine the prevalence of hookworm infections and the pretreatment and posttreatment epg for each group. The associations between hookworm infections and factors such as sex, age, purpose, and settlement were assessed using logistic regression analysis. Paired *t*-tests were used to compare pretreatment and posttreatment counts within the same group and independent *t*-tests to compare treated and untreated groups. Effective IC_50_ values from in vitro study were calculated using logistic model fitting curve (dose–response (inhibition)). All analyses were done using R statistical software version 4.4.1 and GraphPad Prism version 10.1.

### 2.9. Ethical Consideration

Ethical approval was obtained from the Animal Ethics Committee at KNUST (KNUST/AREC/C.1, 0063) before the study began. Informed consent was sought from dog owners prior to sample collection. Animal welfare guidelines were strictly followed during sample collection and treatment. The efficacy tests were done based on recommendations of World Association for the Advancement of Veterinary Parasitology (WAAVP).

## 3. Results

**Figure 2:**
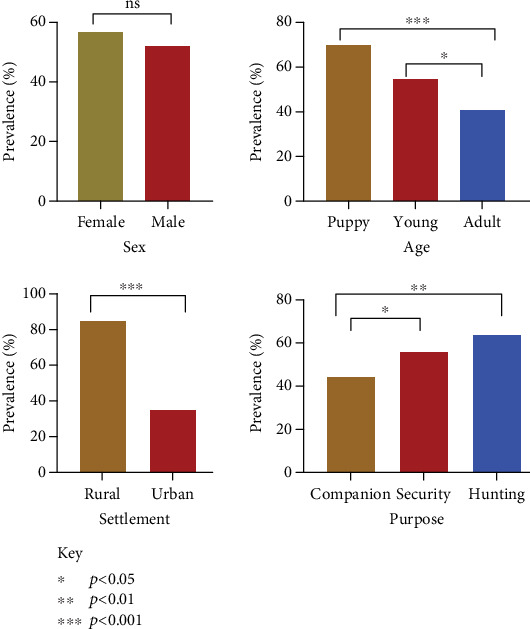
Hookworm prevalence among dogs in Bono East.

**Figure 3:**
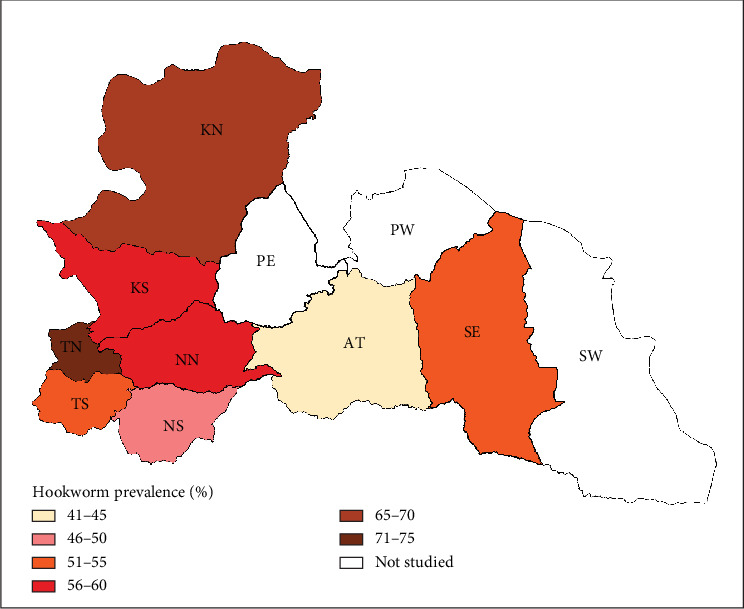
District prevalence of hookworm infection in dogs.

This study surveyed 491 dogs, revealing a hookworm prevalence of 54.2% (266). Age was significantly associated with infection rates (**X**^2^ = 1.001, *p* < 0.00001), with puppies exhibiting the highest prevalence (69.8%), followed by young dogs (55.2%) and adults (41%). The purpose for which dogs were kept also influenced infection rates (**X**^2^ = 12.288, *p* = 0.0021), with hunting dogs showing the highest prevalence (64.1%), followed by security dogs (55.6%) and companion dogs (44.6%). Settlement type was another significant factor, with rural dogs showing higher prevalence (84.2%) compared to urban dogs (35.2%) (Figure 2). The district prevalence ranged from 43% to 74% (Figure 3).

The analysis of risk factors revealed several important associations (Table 2). Puppies were more likely to be infected than adult dogs (adjusted odds ratio (AOR) = 3.625, 95%CI = 0.534–4.421, *p* = 0.003). However, there was no significant difference in infection rates between young dogs and adults (AOR = 2.127, 95%CI = 0.759–2.539, *p* = 0.451). When considering the dogs' purpose, those used for hunting had significantly higher chances of hookworm infection compared to companion dogs (AOR = 1.56, 95%CI = 0.337–1.932, *p* = 0.026). No significant link was found for dogs kept for security purposes (AOR = 0.786, 95%CI = 0.478–1.292, *p* = 0.342). The type of settlement also played a role in infection risk, with dogs in rural areas showing a slightly higher chance of infection than those in urban areas (AOR = 1.519, 95%CI = 0.994–2.321, *p* = 0.05) (Table 2).

**Figure 4:**
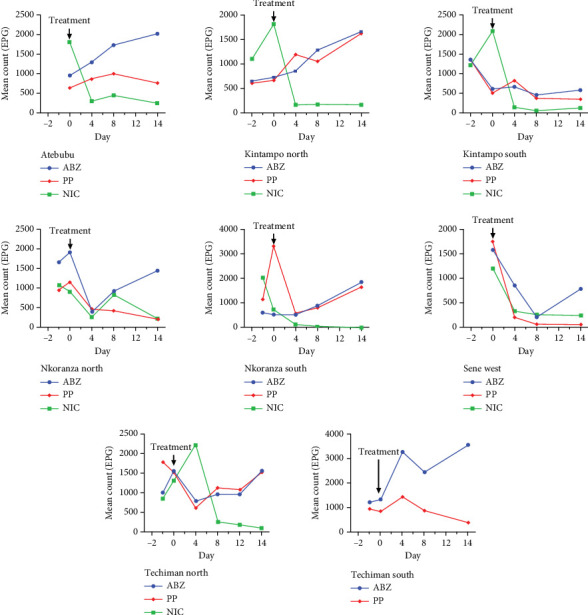
Fecal egg counts taken before and after treatment of dogs with ABZ (albendazole), PP (pyrantel), and NIC (niclosamide) within the eight districts. *(District names are shown on each panel. Arrows indicate the day of treatment (Day 0).)*

**Figure 5:**
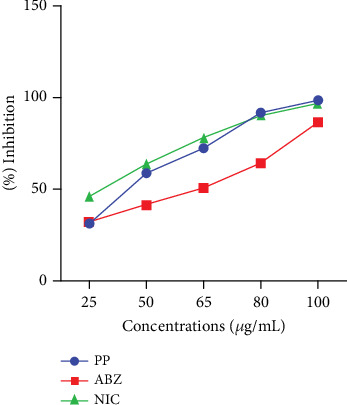
Mean plot of anthelmintic inhibition against different concentrations.

**Figure 6:**
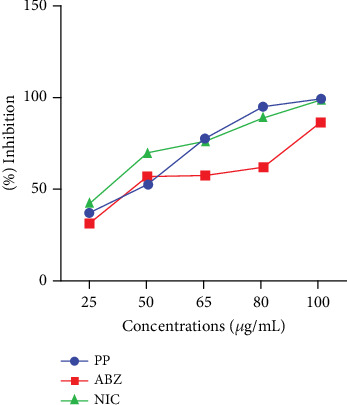
Median plot of anthelmintic inhibition against different concentrations.

This study also evaluated the efficacy of three anthelmintics: ABZ, PP, and NIC, in reducing helminth egg counts in infected dogs (Figure 4). ABZ had a negative efficacy of −69%, as seen by a considerable increase in posttreatment egg counts (Table 2). PP showed a low efficacy of 2.5%, but NIC exhibited a high efficacy of 95% (Table 3). In vitro inhibition percentages of ABZ, PP, and NIC were assessed at various concentrations (100, 80, 65, 50, and 25 *μ*g/mL). PP showed the highest inhibition at 100 *μ*g/mL, followed by NIC at 96.73% (Figures 5 and 6). ABZ showed lower inhibition at 86.49%. NIC consistently demonstrated higher inhibition compared to PP and ABZ at lower concentrations. At 65 *μ*g/mL, NIC showed the highest inhibition (78.16%), followed by PP (72.3%) and ABZ (50.71%). The lowest IC_50_ value for NIC was 29.19 *μ*g/mL, with PP at 38.20 *μ*g/mL and ABZ at 52.41 *μ*g/mL. Higher *R*^2^ values indicated a stronger relationship between drug concentration and observed response (Table 4).

**Figure 7:**
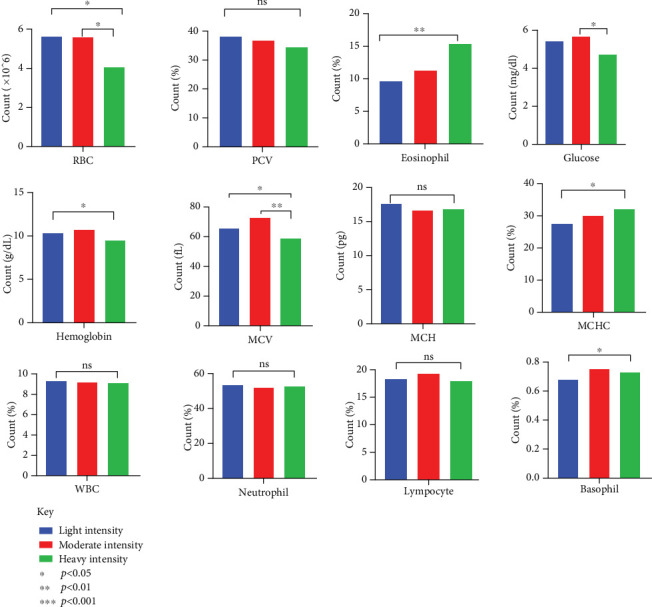
Hematological changes of parameters in canine with varying degrees of infection.

**Figure 8:**
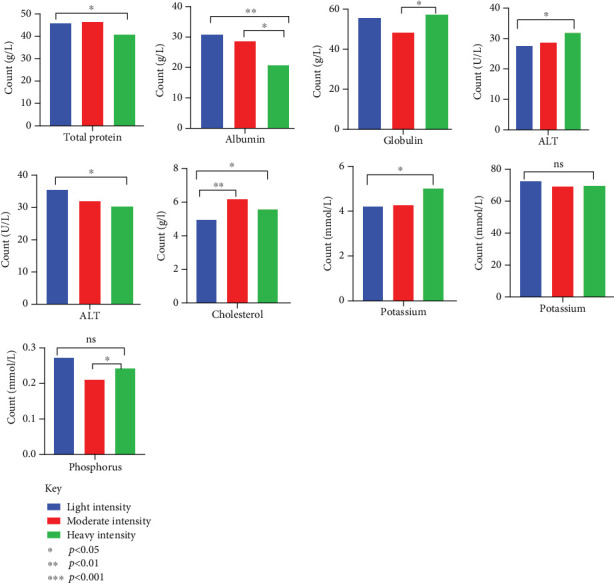
Biochemical changes of parameters in canine with varying degrees of infection.

Dogs that were heavily infected had considerably reduced number of RBCs with an average count of 4.07 ± 0.27, compared to dogs with light infections (average count of 5.63 ± 0.43) and moderate infections (average count of 5.60 ± 0.44) (*p* = 0.025) (Table 5). Infection severity also had an impact on Hb levels and MCV (Figure 7). The percentage of eosinophils increased in correlation with the severity of the infection (*p* = 0.003). There were notable alterations in the biochemical markers, with total protein, albumin, and globulin levels fluctuating with the severity of infection. ALT and AST levels were, however, within reference ranges (Figure 8).

## 4. Discussion

This study found a prevalence of 54.2% among the 491 dogs that were surveyed. This finding highlights a significant health concern in the region. The high prevalence rate reported in this study is consistent with previous studies in Ghana [7–9, 26] and other countries [33–36]. Hookworm prevalence rates vary across regions, with significantly higher rates in Africa due to various factors such as warm climates, poor sanitation, and limited animal healthcare. Lower prevalence of hookworms reported in Europe [33, 37, 38] is likely due to better access to veterinary care and stricter public health measures.

The worldwide extent and persistent nature of the challenge of hookworm infections in dogs necessitate periodic surveillance. In Africa, microscopic examination is the most common method for identifying hookworm infections. However, microscopy has low sensitivity and may potentially lead to underreporting or misidentification of less common or emerging species. The few molecular-based studies in Africa have identified *Necator americanus* [8], *A. caninum* [39], *Ancylostoma braziliense* [5], *Uncinaria stenocephala* [40], and *Ancylostoma ceylanicum* [41] as the species infecting dogs. Although *Ancylostoma ceylanicum* was originally thought to be restricted to Asia and the Pacific regions, it has been reported in Tanzania, Africa. This finding necessitates further molecular surveillance in dogs to elucidate the distribution of hookworm species within the subregion.

Understanding the risk factors for infection is important for creating active control programs. Factors such as age, purpose, location, housing styles, and deworming status can increase the likelihood of dogs becoming infected with parasites [15, 26, 28, 42, 43]. In the present study, puppies exhibited higher infection rates. This is largely due to the lack of acquired immunity and behaviors that increase exposure to contaminated environments. Hunting dogs also showed a high prevalence of hookworm infections, which concurs with Amissah-Reynolds et al., who reported a 72.1% infection rate in hunting dogs [26]. This can be attributed to their frequent exposure to outdoor environments which favor hookworm transmission. Rural areas reported significantly higher hookworm prevalence (84.2%) compared to urban areas (35.2%). Amissah-Reynolds et al. [7] also reported higher prevalence of parasite in poor settlement site. Studies from Ghana, Holland, and Slovakia corroborate the trend of higher parasitic infections in rural settings [44–46]. Higher rural prevalence may be due to more stray dogs contaminating the soil with parasite-infested excrement. This highlights the need for better sanitation and veterinary care in these areas.

Significant changes in blood and biochemical parameters were observed in dogs with moderate and heavy infections. The decrease in RBC count and Hb levels, especially in dogs with heavy infections, is in accordance with previous studies [22, 47, 48]. These changes are due to the blood loss caused by the parasites, which attach to the intestinal wall and feed on the host's blood, leading to anemia [49]. Eosinophilia in heavily infected dogs is a common immune response to parasites, as seen in studies from Kenya and Nigeria [50, 51]. The lower MCV in heavily infected dogs suggests microcytic anemia, caused by iron deficiency from chronic blood loss [23]. This finding suggests the importance of early treatment to avert further complications. Hypoalbuminemia and hypoproteinemia in heavily infected dogs were in concordance with reports in hookworm-infected dogs [52–54]. These conditions are likely due to protein loss through the intestines and reduced production by the liver, as the body is stressed by the infection. Changes in liver enzymes, such as the decrease in ALT and increase in AST, suggest that the liver may be under strain or damaged. This observation aligns with the findings from necropsy studies in Egyptian dogs as reported by Abbas et al. in their systematic review [55]. Investigating detailed eosinophil responses and other immune markers is important. This can enhance treatment plan and contribute to the development of effective vaccines since vaccines are not available for hookworm treatment.

The high prevalence of hookworm infections suggests the need for evaluating the efficacy of available anthelmintics in Ghana. This trial is the first randomized control study comparing the efficacy of ABZ, PP, and NIC in treating hookworm infections in dogs in Ghana. Egg counts increased after ABZ, in all 72 dogs, resulting in a total efficacy of −69%. This increase may be due to density-dependent fecundity, where worms susceptible to ABZ are eliminated, thus allowing female hookworms to produce more eggs [56, 57]. This suggests possible resistance of hookworms to ABZ. This raises concerns in drug resistance, poor drug quality, reinfection, or biological differences among dog populations in the study area. Similarly, ABZ resistance has been reported in dogs [58–60], although high efficacy of ABZ has been reported in ruminants [61–63]. Previous studies in Ghana have isolated *Necator americanus*, a hookworm known to infect humans in dogs [8]. The low efficacy observed in dogs, as well as in humans [64] in the study area, could be linked to the transmission of zoonotic species. Genetic indicators linked to BZ resistance have been identified in hookworm samples from Ghana, indicating that the continual use of ABZ may enhance the presence of mutations associated with resistance [65]. This raises concerns about species-specific efficacy test, calling for treatments tailored to the particular species involved. Low ABZ efficacy could also be attributed to higher average egg count before treatment, since lighter infections tend to respond better to BZs like ABZ than heavier infections [66].

PP is a widely used anthelmintic for treating hookworm in dogs in Ghana [67]. However, widespread reports of PP resistance in canine hookworm in the United States [10, 68–70], Australia [71], and Canada [12] are of concern. The limited efficacy observed could be due to the emergence of drug-resistant strains of hookworms and issues related to under- or overdosage. Prazivet (the PP-based drug) used in this study is a broad-spectrum anthelmintic that contains PP pamoate, praziquantel, and febantel. The primary activity against hookworms comes from PP pamoate, with praziquantel and febantel effective against cestodes [72] and whipworms, respectively [14]. The low efficacy observed with Prazivet in vivo indicates reduced susceptibility of hookworms to PP. The higher PP efficacy observed in the in vitro assay could be attributed to suboptimal drug absorption or rapid metabolism [68]. These factors emphasize the necessity of integrating both in vitro and in vivo data when assessing the effectiveness of anthelmintic.

NIC showed higher efficacy in the current study compared to ABZ and PP. While high efficacy of NIC against cestodes and *Toxoplasma gondii* has been reported in pets [73], there is limited information on the efficacy of this drug against hookworms. The mechanism of action of NIC involves disrupting the parasite's metabolic processes, making it very effective compared to other anthelmintics [73]. The rare use of NIC in the area may be influencing the development of resistance in this local population. These results suggest that NIC could be a strong alternative for treating hookworm infections, especially where traditional treatments have failed. Further research and clinical trials are needed to confirm these findings. This will help determine whether NIC should be included in routine treatment plans for canine hookworm infections.

The efficacy of NIC and PP in treating hookworm infections was demonstrated by significant improvements in Hb levels, nutritional status, and overall metabolic health. These findings are consistent with studies by Jarry and Alfatlawi, Kryvoruchenko, Silalahi et al., and De et al., who also reported marked hematological recovery following the use of effective anthelmintics [21, 74–76]. The strong immune response and reduced inflammation observed in the NIC and PP groups further indicate their success in combating hookworm-induced anemia. In contrast, ABZ treatment groups showed only moderate improvements, with limited gains in blood and protein levels and a small reduction in inflammation. This aligns with previous research, which noted that ABZ has variable efficacy depending on the severity of the infection and the parasite's resistance levels [76].

## 5. Conclusion

The study found an overall prevalence of 54.2% (266), with age, purpose, and settlement identified as risk factors for hookworm infections. NIC (95%) proved highly efficacious in reducing hookworm infections. The low efficacy of PP (2.5%) and ABZ (−69%) suggests anthelmintic resistance. Hookworm-infected dogs exhibited anemia, eosinophilia, hypoalbuminemia, and hypoproteinemia. The improvement in hematological and biochemical parameters posttreatment highlights the health benefits of effective anthelmintic intervention. It is recommended that the efficacy of ABZ, PP, and NIC should be tested in different regions of Ghana to capture the overall efficacy of these drugs in Ghana.

## Figures and Tables

**Table 1:** Treatment group for the experimental control trial.

**Treatment**	**Number of dogs**
Albendazole (ABZ)	76
Pyrantel (PP)	76
Niclosamide (NIC)	70
Placebo	27

**Table 2:** Risk factors of hookworm infection among dogs.

**Predictor**	**Adjusted odds ratio (AOR)**	**95% CI AOR**	**p** ** value**
*Age*			
Adult	Reference		
Young	2.127	0.759, 2.539	0.451
Puppies	3.625	0.534, 4.421	0.003
*Purpose*			
Companion	Reference		
Security	0.786	0.478, 1.292	0.342
Hunting	1.56	0.337, 1.932	0.026
*Settlement*			
Urban	Reference		
Rural	1.519	0.994, 2.321	0.05

**Table 3:** In vivo efficacy of anthelmintic in reducing helminth egg counts in dogs.

**Anthelmintic**	**Location**	**No. of dogs**	**Egg count before treatment (days before/Day 0)**	**Pretreatment**	**Egg count after treatment (days after Day 0)**	**Posttreatment**	**Efficacy (%)**
ABZ	AT	11	0	642 ± 67.3	4, 8, 14	876 ± 112.1	−36
	KN	10	−2, 0	685 ± 93.0	4, 8, 14	1265 ± 190.6	−85
	KS	6	−2, 0	490 ± 59.5	4, 8, 14	569 ± 57.4	−16
	NN	11	−1, 0	784 ± 195.7	4, 8, 14	930 ± 113	−19
	NS	8	−1, 0	1580 ± 66.1	4, 8, 14	1098 ± 161.6	31
	SW	10	0	575 ± 82.1	4, 8, 14	613 ± 119.6	−7
	TN	10	−1, 0	679 ± 74.2	4, 8, 14	1163 ± 220.6	−71
	TS	10	−1, 0	2286 ± 127.1	4, 8, 14	3098 ± 605.5	−36
	Total	76	−2 to 0	730 ± 46.7	4–14	1229 ± 125.8	−69

PP	AT	9	0	963 ± 84.3	4, 8, 14	1679 ± 355.6	−74
	KN	10	−2, 0	632 ± 64.2	4, 8, 14	1288 ± 194.4	−104
	KS	6	−2, 0	446 ± 47.3	4, 8, 14	518 ± 44.1	−16
	NN	9	−1, 0	1054 ± 136.6	4, 8, 14	372 ± 89.7	65
	NS	10	−1, 0	2247 ± 454.5	4, 14	1015 ± 186.5	55
	SW	9	0	744 ± 201.6	4, 8, 14	106 ± 34.5	86
	TN	13	−1, 0	567 ± 114.2	4, 8, 14	1248 ± 346.5	−120
	TS	10	−1, 0	908 ± 79.1	4, 8, 14	917 ± 217.8	−1
	Total	76	−2 to 0	957 ± 91.9	4–14	934 ± 100	2.5

NIC	AT	12	0	1808 ± 178.5	4, 8, 14	65 ± 11.7	96
	KN	10	−2, 0	1460 ± 196.5	4, 8, 14	115 ± 22.2	92
	KS	10	−2, 0	2808 ± 112.1	4, 8, 14	85 ± 14.6	96
	NN	7	−1, 0	1494 ± 203.4	4, 8, 14	148 ± 17.7	90
	NS	10	−1, 0	1390 ± 210.3	4, 8, 14	48 ± 11.7	96
	SW	10	0	1192 ± 136.1	4, 8, 14	76 ± 27.7	94
	TN	11	−1, 0	762 ± 167.5	4, 8, 14	42 ± 7.4	94
	Total	70	−2 to 0	1559 ± 68.2	4–14	82 ± 18.5	95

Abbreviations: ABZ, albendazole; AT, Atebubu; KN, Kintampo North; KS, Kintampo South; NIC, niclosamide; NN, Nkoranza North; PP, Prazivet Plus; SW, Sene West; TN, Techiman North; TS, Techiman South.

**Table 4:** Comparative IC_50_ values of anthelmintic for treating hookworm infections.

**Anthelmintic**	**IC**_**50**_	**R** ^2^	**95% CL**
Albendazole	52.41	0.7306	29.79–72.37
Pyrantel	38.2	0.9870	27.29–48.62
Niclosamide	29.19	0.9941	15.30–39.52

**Table 5:** Hematobiochemical parameters of different treatment groups on Days 0 and 14.

	**ABZ**		**PP**		**NIC**		**Placebo**		**Healthy control**	
	**Day 0**	**Day 14**	**Day 0**	**Day 14**	**Day 0**	**Day 14**	**Day 0**	**Day 14**	**Day 0**	**Day 14**
*Parameters*										
RBC (× 10^6^)	5.36 ± 0.48	6.14 ± 0.18	5.16 ± 0.46	6.44 ± 0.33	5.08 ± 0.030	6.30 ± 0.24	5.21 ± 0.70	4.89 ± 0.55	6.90 ± 0.53	6.06 ± 0.51
Hb (g/dL)	8.44 ± 0.50	12.25 ± 0.68^∗^	10.21 ± 1.02	13.11 ± 1.37^∗^	7.42 ± 0.40	14.76 ± 0.86^∗^	10.61 ± 1.07	9.46 ± 0.97	11.87 ± 1.35	12.56 ± 1.06
PCV (%)	35.29 ± 1.51	38.70 ± 1.53	33.51 ± 3.13	38.49 ± 2.54	31.49 ± 2.3.	43.99 ± 1.69^∗^	32.61 ± 3.04	35.45 ± 2.53	39.86 ± 0.99	42.67 ± 3.18
MCV (fL)	58.69 ± 3.23	60.50 ± 307	65.01 ± 4.52	58.86 ± 4.71	59.42 ± 4.52	50.99 ± 5.45	71.15 ± 1.76	71.88 ± 1.84	77.31 ± 3.83	59.46 ± 5.42
MCH (pg)	18.34 ± 1.24	20.86 ± 1.45	20.39 ± 1.04	25.59 ± 2.06	16.28 ± 1.95	17.19 ± 2.04	23.34 ± 1.55	24.55 ± 1.71	11.84 ± 1.23	12.97 ± 1.45
MCHC (%)	29.64 ± 1.15	32.07 ± 1.25	31.29 ± 2.16	36.63 ± 1.91	33.16 ± 1.29	34.55 ± 1.54	34.09 ± 0.84	35.74 ± 1.62	27.88 ± 2.44	28.48 ± 4.07
WBC (× 10^3^)	10.34 ± 0.46	12.42 ± 0.64	7.11 ± 0.46	12.31 ± 0.80	12.29 ± 1.01	13.81 ± 0.92	11.41 ± 0.65	12.96 ± 0.88	8.13 ± 1.07	8.60 ± 1.21
Neutrophil (%)	44.56 ± 3.17	47.09 ± 2.64	56.62 ± 3.06	58.17 ± 2.88	39.62 ± 2.28	44.96 ± 3.14	63.02 ± 4.66	63.44 ± 4.06	49.13 ± 5.17	48.45 ± 4.55
Monocyte (%)	3.72 ± 0.28	6.68 ± 0.30	6.87 ± 0.63	8.34 ± 0.75	6.36 ± 0.60	8.36 ± 0.44	6.09 ± 0.66	7.55 ± 0.43	9.39 ± 0.75	10.53 ± 0.86
Lymphocyte (%)	26.94 ± 3.16	28.34 ± 3.16	14.11 ± 0.66	15.51 ± 0.66	17.91 ± 1.07	19.31 ± 1.07	19.42 ± 2.62	20.82 ± 2.62	20.65 ± 4.56	22.05 ± 4.56
Eosinophil (%)	10.54 ± 0.77	11.04 ± 0.77	10.03 ± 1.00	11.53 ± 1.00	9.91 ± 0.61	16.41 ± 0.61^∗^	8.52 ± 0.96	10.02 ± 0.96	10.40 ± 1.16	11.90 ± 1.16
Basophil (%)	0.49 ± 0.05	1.19 ± 0.05	0.86 ± 0.17	1.56 ± 0.17	0.99 ± 0.21	1.69 ± 0.21	0.72 ± 0.06	1.42 ± 0.06	0.71 ± 0.05	1.41 ± 0.05
*Biochemical*										
Total protein (g/L)	55.09 ± 4.92	59.79 ± 3.75	31.64 ± 2.79	56.07 ± 2.82^∗^	44.02 ± 1.87	63.35 ± 1.29^∗^	33.20 ± 2.41	31.16 ± 2.92	57.18 ± 3.28	58.20 ± 3.70
Albumin (g/L)	27.70 ± 1.02	35.13 ± 0.60	27.60 ± 1.13	36.43 ± 2.46^∗^	26.61 ± 0.49	37.77 ± 0.88^∗^	25.44 ± 1.49	28.54 ± 0.13	33.52 ± 0.75	35.92 ± 1.25
Globulin (g/L)	56.12 ± 4.56	36.35 ± 0.92^∗^	62.99 ± 6.29	37.03 ± 1.72^∗^	56.29 ± 3.29	39.11 ± 1.88^∗^	52.56 ± 5.79	50.16 ± 2.23	34.32 ± 0.57	30.76 ± 2.50
Glucose (mg/dL)	5.45 ± 1.17	5.85 ± 0.27	4.77 ± 0.39	6.77 ± 0.40	6.06 ± 0.45	7.13 ± 0.36	6.50 ± 0.20	6.96 ± 0.30	5.74 ± 0.23	4.98 ± 0.32
Cholesterol (mmol/L)	5.95 ± 0.25	5.87 ± 0.27	4.77 ± 0.42	6.14 ± 0.52	4.33 ± 0.31	4.97 ± 0.37	6.54 ± 1.03	5.52 ± 0.35	6.22 ± 0.40	7.84 ± 0.15
ALT (IU/L)	30.35 ± 3.30	31.22 ± 3.08	43.73 ± 4.18	45.60 ± 4.08	37.06 ± 3.91	38.40 ± 4.58	33.76 ± 6.48	27.42 ± 5.81	14.98 ± 0.54	23.34 ± 5.74
AST (IU/L)	26.50 ± 2.73	32.78 ± 2.69	23.49 ± 3.32	27.73 ± 3.44^∗^	39.20 ± 3.54	47.37 ± 3.29^∗^	28.46 ± 3.07	35.06 ± 3.61	34.16 ± 5.00	34.88 ± 4.51
Creatinine (*μ*mol/L)	72.75 ± 5.84	90.20 ± 8.48^∗^	66.97 ± 5.58	68.00 ± 3.58	84.73 ± 3.30	84.17 ± 3.80	76.18 ± 4.58	72.52 ± 4.59	45.52 ± 8.36	51.08 ± 3.69
Phosphorus (mmol/L)	0.25 ± 0.03	1.45 ± 0.04	0.30 ± 0.05	1.60 ± 0.06	0.21 ± 0.03	1.41 ± 0.07	0.16 ± 0.03	1.28 ± 0.07	0.26 ± 0.05	0.98 ± 0.19
Potassium (mmol/L)	4.13 ± 0.52	4.82 ± 0.16	4.60 ± 0.55	5.47 ± 0.47	6.03 ± 0.70	5.67 ± 0.36	2.96 ± 0.50	4.48 ± 0.35	4.52 ± 0.45	4.38 ± 0.42

*Note:* Placebo—infected but not treated, healthy dogs—dogs which were not infected.

Abbreviations: ABZ, albendazole; NIC, niclosamide; PP, pyrantel.

⁣^∗^Observed significant difference.

## Data Availability

The data supporting the findings of this study are available from the corresponding author upon reasonable request.
